# Society Meetings

**Published:** 1898-04

**Authors:** 


### Society Meetings.

The Niagara Falls Academy of Medicine at its regular monthly meeting, held March 7, 1898, adopted the following preambles and resolutions relating to the osteopathy bill:

Whereas, A bill has been recently introduced in the state legislature by Mr. Raplee, regulating and legalising the practice of osteopathy in the state of New York ; and

Whereas, Said bill provides that the so-called science of osteopathy may be practised by individuals without examination or other test as to their fitness to practise any method of treatment of disease ; and

Whereas, Section 5 of this bill provides as follows : "The system, method or science of treating diseases of the human body, commonly known as 'osteopathy,' is hereby declared not to be the practice of medicine within the meaning of Chapter 661 of the laws of 1893, entitled 'An act in relation to the public health, constituting Chapter 25 of the general laws,' and is not subject to the provisions of said chapter or to the laws amendatory thereof." Therefore be it

*Resolved*, That as it has already been held by high authority that any method which aims at the cure of disease practised by any person, is legally the practice of medicine. Be it

*Resolved*, That the Niagara Falls Academy of Medicine does hereby declare that the practice of osteopathy, so-called, is an attempt to practise the science and art of medicine. Be it further

*Resolved*, That any attempt to legalise the practice of osteopathy without submitting the applicants to the same examination and test as to their efficiency and ability to practise medicine by any other method, is a direct blow at the high standard reached in this state by the passage of Chapter 661 of the laws of 1893, entitled "An act in relation to the public health, constituting Chapter 25 of the general laws," and is a step backward. Be it further

*Resolved*, That it is the sense of this academy of medicine that the laws for the protection of public health and the practice of medicine in general should be made more stringent instead of allowing any loophole whereby the public may be imposed on by unscrupulous persons. Be it further

*Resolved*, That this academy request the representatives of this district in Senate and Assembly to use all honorable means to prevent the passage of the aforesaid mentioned bill.

Dr. Guillemont read a paper on Locomotor ataxia, its recent pathology and treatment. After this was discussed and some routine business transacted the Academy adjourned.

The Buffalo Academy of Medicine held meetings during the month of March, 1898, as follows :

*Section on Surgery.*—Tuesday evening, March 1st, program : Parasitism in skin diseases, by Dr. F. J. Thornbury ; Presentation of case of intraorbital tumor, by Dr. E. E. Blaauw ; Exhibition of specimens, by Drs. H. E. Hayd and C. P. Smith.

*Section on Medicine.*—Tuesday evening, March 8th, program : The relation of bacteria to the normal alimentary canal, by Dr. Herbert U. Williams ; Cardio-pulmonary murmurs, by Dr. C. F. Hoover, professor of physical diagnosis, medical department, Western Reserve University, Cleveland, O.

*Section on Pathology.*—Tuesday evening, March 15th, program : The anatomy and mechanism of the foot considered with reference to certain abnormal conditions, by Dr. L. A. Weigel, Rochester, N. Y. Illustrated by stereopticon.

*Section on Obstetrics and Gynecology.*—Tuesday evening, March 22d, program : Etiology, pathology, diagnosis and treatment of acute salpingitis, by Dr. Wm. G. Taylor.

---

The Association of Surgeons of the Southern Railway Company will hold its third annual meeting at the Hygeia Hotel, Old Point Comfort, Virginia, Tuesday and Wednesday, June 21 and 22, 1898, under the presidency of Dr. C. M. Drake, of Atlanta. Several distinguished surgeons from New York, Philadelphia and other cities are expected to be present and address the meeting. A printed program will be issued about June 10th. The Hygeia Hotel has made a special rate of $2.50 a day for members and guests of the association. Correspondence relating to the meeting should be addressed to Dr. T. H. Hancock, secretary, Atlanta, Ga.

---

The National Confederation of State Medical Examining and Licensing Boards will hold its eighth annual meeting at Denver, Monday, June 6, 1898. A number of interesting papers will be presented relating to state control in medicine and a report by the committee on preliminary requirements for entrance to medical study will be presented. Correspondence relating to the meeting should be addressed to Dr. A. Walter Suiter, secretary, Herkimer, N. Y. The sessions are open to all persons interested in the subjects discussed.

The American Medical Association will hold its fifty-first annual meeting at Denver, June 7–10, 1898, under the presidency of Brigadier-General George M. Sternberg, M. D., surgeon-general of the U. S. army. Preparations for the meeting are well advanced, hotel accommodations are ample and reasonable and everything portends a large and interesting meeting.

---

The American Electro-therapeutic Association will hold its seventh annual meeting at Buffalo, September 13, 14 and 15, 1898, under the presidency of Dr. Charles R. Dickson, of Toronto. The titles of papers to be read at the meeting should be furnished to Dr. John Gerin, secretary, 68 North street, Auburn.

---

The American Academy of Medicine, as announced in our March edition, (q. v.) will hold its twenty-third annual meeting at Denver, June 4 and 6, 1898. Those who desire to join an excursion of the Fellows to Denver should communicate with Dr. Charles McIntire, secretary, Easton, Pa.

---

The American Medical Publishers' Association will hold its fifth annual meeting at Denver, Col., Monday, June 6, 1898. Contributors who desire to be heard at the meeting should send titles of their papers at once to Charles Wood Fassett, secretary, St. Joseph, Mo.

